# Alternate-Day Fasting Ameliorates Newly Established Sjögren’s Syndrome-like Sialadenitis in Non-Obese Diabetic Mice

**DOI:** 10.3390/ijms232213791

**Published:** 2022-11-09

**Authors:** Dongfang Li, Shoko Onodera, Shu Deng, Bashaer Alnujaydi, Qing Yu, Jing Zhou

**Affiliations:** The Forsyth Institute, 245 First Street, Cambridge, MA 02142, USA

**Keywords:** Sjögren’s syndrome, alternate-day fasting, salivary secretion, leukocyte infiltration

## Abstract

Intermittent fasting confers protections to various diseases including autoimmune disorders, but the specific effects of intermittent fasting on Sjögren’s syndrome (SS) remains inconclusive. The present study was undertaken to determine the specific impact of alternate-day fasting (ADF) on newly established SS-like sialadenitis using non-obese diabetic (NOD) mice. Female NOD mice were deprived of food every other day from 10 to 13 weeks of age, the early stage of established SS, and then analyzed for the disease characteristics. Mice in the ADF group had higher salivary flow rate and attenuated submandibular gland (SMG) inflammation, compared to the control mice fed with standard chow ad libitum. The improvements were accompanied with a decrease in the total leukocytes, T and B lymphocytes and activated CD4 and CD8 T cells, and a down-regulation of pro-inflammatory cytokines IFN-γ and IL-17, chemokine receptor CXCR3 and its ligands CXCL9 and CXCL11 in the SMGs. ADF also led to elevated mRNA levels of water channel protein aquaporin 5 and tight junction protein claudin-1, two factors crucial for normal salivary secretion in the SMGs. In addition, ADF reduced the proportion of IFN-γ- and IL-17- expressing CD4 T cells and diminished mRNA levels of IFN-γ, TNF-α, and IL-17 in the total submandibular draining lymph node cells. Taken together, ADF is effective in ameliorating newly established SS-associated salivary gland exocrinopathy in NOD mice.

## 1. Introduction

Sjögren’s Syndrome (SS) is a systemic autoimmune disease affecting 2–4 million Americans [1]. In this disorder, immune system primarily attacks salivary and lacrimal glands, causing tissue inflammation and damage, secretory hypofunction and autoantibody production [1,2,3,4]. The most common symptoms of SS are dry mouth and dry eyes [1,2]. Although the etiology of SS is largely unknown, accumulating evidence has shown that T and B cells comprise the majority of infiltrates in exocrine glands and crucially contribute to the development and progression of this disease by secreting pro-inflammatory cytokines and producing auto-antibodies to damage disease-target organs and impair their secretory function [5,6,7,8]. In patients with SS, the levels of IFN-γ and IL-17, signature cytokines of T helper (Th)1 and Th17 cells are elevated in salivary glands and highly correlated with the disease severity [1,9,10]. Studies with mouse models also demonstrated the indispensable pathogenic role of IFN-γ and IL-17 in the development and onset of SS [11,12,13,14]. In addition to the pro-inflammatory cytokines produced by effector T cells, T cell trafficking into the disease-target salivary glands is also a vital pathogenic event of SS, which requires the interaction of T cell-expressed chemokine receptors, such as CXCR3 and their specific ligands CXCL9, CXCL10 and CXCL11 [15]. Moreover, the expression of these T cell chemoattractants CXCL9 and CXCL10 in salivary gland tissues can also be induced by IFN-γ, further contributing to recruitment of CXCR3-expressing T cells and gland damage [1,16]. Consistent with these lines of evidence, we and other groups previously reported that inhibition of CXCR3 function or antagonism of its ligands significantly impeded the development and onset of SS-like sialadenitis and xerostomia by reducing effector T cell infiltration of SMGs in SS mouse models [17,18]. Hence, approaches that can reduce T and B cell infiltrations and/or dampen autoreactive T and B cell responses in salivary gland may serve as therapeutic strategy for SS.

Intermittent fasting, defined as recurrent prolonged periods of food deprivation but with unrestricted access to water, has been shown to have a profound beneficial impact on various diseases, including autoimmune and inflammatory diseases, in human and animal models [19,20,21]. Laboratory research has demonstrated that intermittent fasting or a fasting-mimicking diet causes a reduction in disease clinical severity and amelioration of disease progression in experimental autoimmune encephalomyelitis, multiple sclerosis, and inflammatory bowel disease, with these improvements associated with decreased levels of pro-inflammatory cytokines, and reduced effector T cells and other pathogenic immune cells in target tissues using mouse models of these diseases [22,23,24,25,26]. In SS, caloric restriction leads to decreased mRNA levels of IFN-γ, IL-10 and IL-12 in submandibular glands (SMGs) of the autoimmune prone (NZB×NZW) F1 mice [27], suggesting an immunomodulatory impact of dietary restriction on salivary gland inflammation. Here, we investigated the impact of alternate-day fasting (ADF) on SS-like exocrinopathy in NOD mice, a well-defined mouse model that recapitulates human SS disorder. The results reveal a protective effect of ADF against sialadenitis and xerostomia and delineate the underlying molecular and cellular mechanisms.

## 2. Results

### 2.1. ADF Improves SS-Associated Hyposalivation and Mitigates Salivary Gland Inflammation in NOD Mice

**Figure 1:**
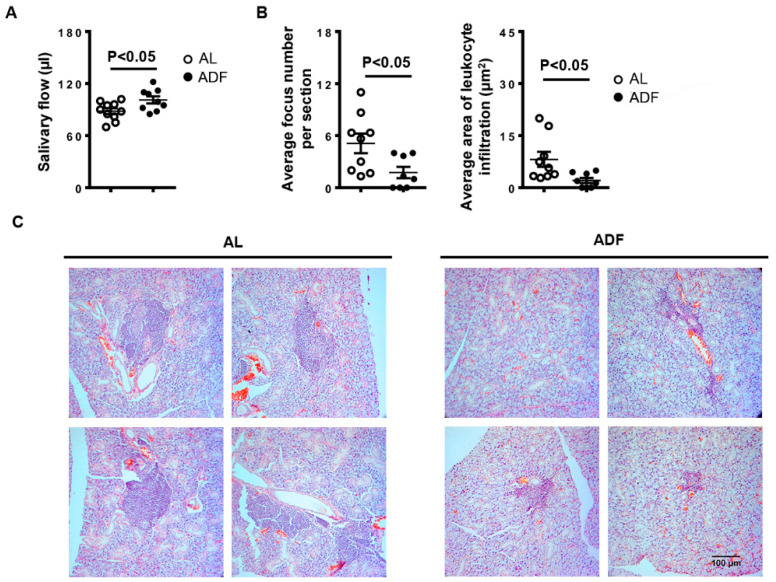
Alternate-day fasting (ADF) improves SS-associated hyposalivation and mitigates salivary gland inflammation in NOD mice. 10-week-old female NOD mice were deprived of food every other day or fed with standard chow ad libitum (*AL*) for 3 weeks. (**A**) The stimulated salivary flow rate. (**B**) The graph shows the average leukocyte focus number and infiltration areas of SMG sections. (**C**) Tile mages of H&E stained areas of one representative SMG sample each treatment group (scale bar = 100 µm). Data are representative or the average of 9–10 mice from each group.

Impaired secretory function and tissue inflammation of salivary glands are the hallmark manifestations of SS [1,2]. To determine the effect of ADF on newly established SS, NOD mice aged 10 weeks received ADF for 3 consecutive weeks or fed standard chow ad libitum, and were analyzed for SS-associated hyposalivation and sialadenitis at 13 weeks old. Mice in ADF group exhibited higher salivary flow rate compared to the control group, suggesting the improvement of salivary secretory function in this SS model (Figure 1A). H&E staining of SMG sections showed significantly lower leukocyte focus numbers and infiltration areas in the mice with ADF than the control mice (Figure 1B,C). Thus, these findings indicate a protective impact of ADF on salivary gland hypofunction and sialadenitis in NOD mice during the early stage of SS establishment.

### 2.2. ADF Decreases T and B Cell Accumulation and Up-Regulates Water Channel Protein Aquaporin 5 (AQP5) and Tight Junction Protein Claudin-1 in the SMGs

**Figure 2:**
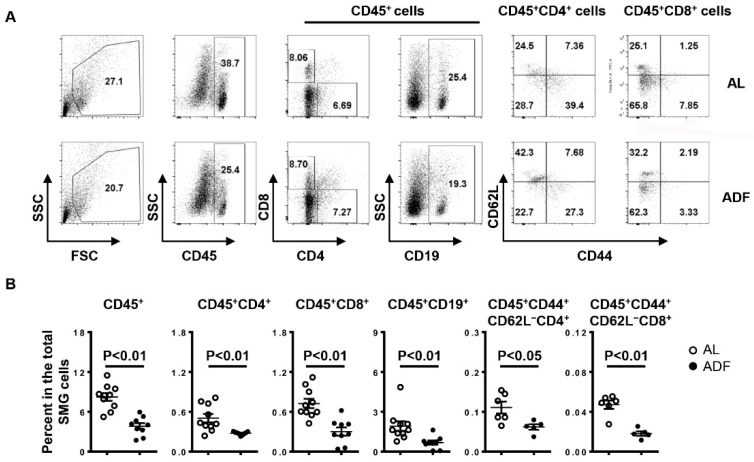
Alternate-day fasting (ADF) decreases T and B cell accumulation in the SMGs. 10-week-old female NOD mice were deprived of food every other day or fed with standard chow ad libitum (*AL*) for 3 weeks. (**A**) Flow cytometry profile of lymphocyte populations in the SMG cells. (**B**) The average percentage of lymphocyte subpopulations among the SMG cells. Data are representative or the average of at least 5–6 mice each group, with majority of the data generated from 7–10 mice each from group.

**Figure 3:**
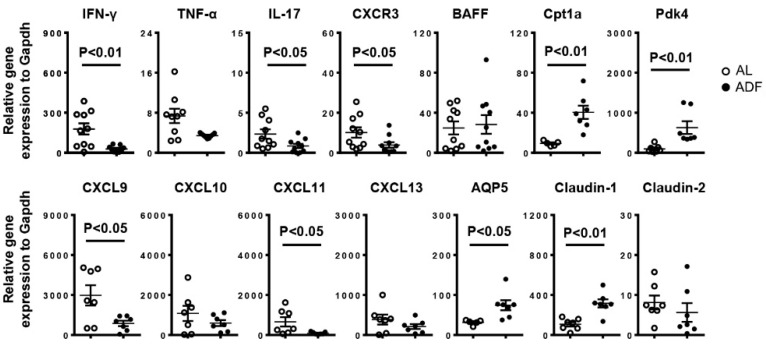
Alternate-day fasting (ADF) down-regulates tissue inflammation-associated genes and up-regulates PPAR pathway target genes, water channel protein AQP5 and tight junction protein claudin-1 in the SMGs. 10-week-old female NOD mice were deprived of food every other day or fed with standard chow ad libitum (*AL*) for 3 weeks. Real-time qPCR analysis of tissue inflammation-associated genes, chemoattractants, PPAR pathway target genes and salivary gland secretion-related factors, presented relative to that of GAPDH. Data are the average 7–10 mice from each group.

We next assessed the effect of ADF on lymphocyte composition in the SMGs of NOD mice. Consistent with the reduced leukocyte focus number demonstrated by H&E staining, flow cytometric analysis showed a significant reduction in the percentage of leukocytes (CD45^+^), CD4 T cells (CD45^+^CD4^+^) and CD8 T cells (CD45^+^CD8^+^) and B cells (CD45^+^CD19^+^) and the frequency of activated CD4 T cells (CD45^+^CD44^+^CD62L^−^CD4^+^) and CD8 T cells (CD45^+^CD44^+^CD62L^−^CD8^+^) among the total SMG cells of NOD mice by ADF (Figure 2A,B). Accordingly, real-time PCR results revealed that ADF markedly reduced the mRNA levels of T helper (Th) 1, T cytotoxic (Tc1) and Th17 cells signature cytokines IFN-γ and IL-17, chemokine receptor CXCR3 and its ligands CXCL9 and CXCL11 in the SMGs (Figure 3). Meanwhile, ADF also up-regulated the total mRNA amounts of PPARγ pathway target genes Cpt1a and Pdk4, water channel protein AQP5 and tight junction protein claudin-1 (Figure 3).

### 2.3. ADF Decreases the Proportion of Th1 and Th17 Cells without Affecting Total CD4 T-, CD8 T Cells and B Cells in the smLNs

**Figure 4:**
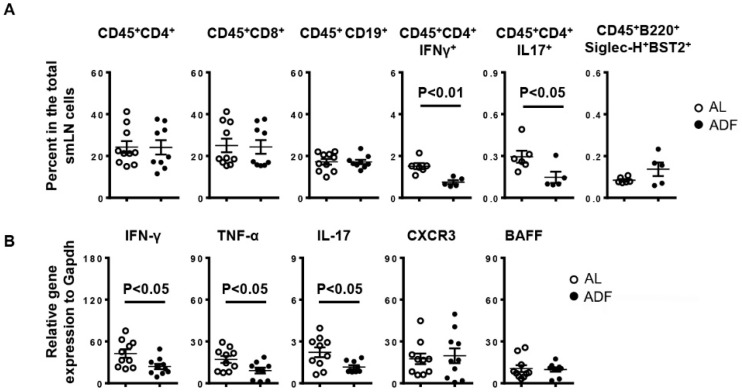
Alternate-day fasting (ADF) decreases the proportion of Th1 and Th17 cells without affecting that of total CD4 T-, CD8 T cells and B cells in the smLNs. 10-week-old female NOD mice were deprived of food every other day or fed with standard chow ad libitum (AL) for 3 weeks. (**A**) The average percentage of lymphocyte subpopulations among the smLN cells. (**B**) Real-time PCR analysis of T and B cell-associated genes, presented relative to that of GAPDH. Data are representative or the average of at least 5–6 mice each group, with majority of the data generated from 9–10 mice from each group.

We next investigated the effect of ADF on lymphocyte subsets in the smLNs by flow cytometry. NOD mice with ADF exhibited a lower percentage of Th1 cells (CD45^+^CD4^+^IFN-γ^+^) and Th17 cells (CD45^+^CD4^+^Th17^+^) among total smLN cells than control mice (Figure 4A). In comparison, the frequency of total CD4 T cells, CD8 T cells and B cells in the total smLN cells were not altered by ADF in a statistically significant manner (Figure 4A). In addition, the proportion of plasmacytoid dendritic cells (CD45^+^B220^+^Siglec-H^+^BST2^+^), a subset of dendritic cells that play a critically pathogenic role in SS as demonstrated by our recent study [28], was slightly increased by ADF, but the change did not reach statistical significance (Figure 4A). Accompanying the reduction in Th1 and Th17 cells, ADF also down-regulated the mRNA levels of IFN-γ, TNF-α and IL-17 in the smLNs (Figure 4B), indicating mitigation of local autoimmune responses. Collectively, ADF ameliorates Th1 and Th17 responses in the smLNs of NOD mice during the early stage of SS establishment.

### 2.4. ADF Does Not Significantly Affect Autoantibody Production in the Serum

**Figure 5:**
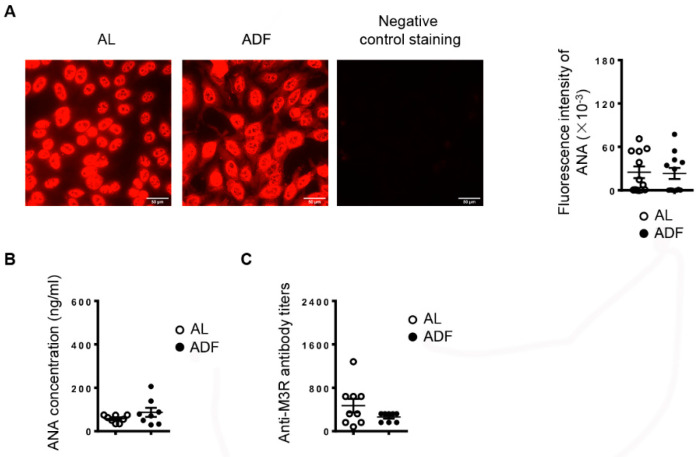
Alternate-day fasting (ADF) does not significantly affect autoantibody production in the serum. 10-week-old female NOD mice were deprived of food every other day or fed with standard chow ad libitum (*AL*) for 3 weeks. (**A**) Representative images of serum ANA detection (scale bar = 50 µm). The graph shows the average fluorescence intensity of ANA staining. (**B**) ELISA analysis of serum ANA concentration. (**C**) ELISA analysis of serum anti-M3R autoantibody titers. Data are representative or the average of 9–12 mice each group.

In addition to secretory hypofunction and tissue inflammation of salivary glands, we further evaluated the impact of ADF on serum autoantibody levels, another clinical characteristic of patients with SS, in NOD mice. By indirect immunofluorescence staining with human Hep-2 epithelial cells as substrates, we found that mice with ADF had a comparable level of serum antinuclear antibodies (ANA) to the control mice (Figure 5A). Consistent with these results, the concentration of the serum ANA from mice with ADF does not significantly differ from that of control group, as determined by ELISA (Figure 5B). Moreover, the titers of autoantibodies against M3R in the sera were not significantly altered by ADF, as demonstrated by enzyme-linked immunosorbent assay (Figure 5C). Hence, ADF provides protection from SS-associated salivary disorder, without affecting serum autoantibody production in NOD mice with established SS.

## 3. Discussion

The present study demonstrated a protective impact of ADF on newly established SS-associated salivary gland inflammation and secretory hypofunction using the NOD mouse model, and dissected the potential mechanisms including a reduction in T and B cell infiltration, mitigation of Th1 and Th17 cell responses and PPAR pathway activity and up-regulation of claudin-1 and AQP5 gene expression in the salivary tissues.

We found that accompanying the decrease in lymphocyte accumulation in the SMGs, ADF also down-regulated T cell chemoattractants CXCL9, CXCL11 and its receptor CXCR3, without significantly altering B cell chemoattractant CXCL13, suggesting the reduction in T cells in the SMGs is attributed to defective cell migration mediated by chemoattraction. The mechanisms by which ADF affects B cell accumulation in the SMG awaits further elucidation.

Apart from cell recruitment, effector T cell-mediated immune responses can also be dampened by intermittent fasting in multiple inflammatory and autoimmune disorders [23,24]. In the salivary tissues of NOD mice, we detected a significant down-regulation of IFN-γ and IL-17, two factors crucial for the pathogenesis and persistence of SS, demonstrated by ours and other groups previous work [11,12,13,14,29,30]. This alteration was paralleled with lowered proportion of activated CD4 and CD8 T cells in the SMGs and Th1 and Th17 cells in the smLNs. Hence, the decreased level of IFN-γ and IL-17 is, at least in part, a consequence of reduced Th1 and Th17 cells. It is also possible that the inflammatory responses of salivary gland resident cells, such as epithelial cells, were mitigated by ADF, contributing to the attenuation of sialadenitis. Further investigation is required to examine this potential cellular mechanism. In addition, ADF did not significantly alter the mRNA level of BAFF, a factor critical for the maturation and survival of B cells in the SMGs, and did not affect the production of antinuclear and anti-M3R autoantibodies, indicating that the protective role of ADF in sialadenitis in NOD mice is not associated with the reduced autoantibody-secreting activities of B cells. Despite the profound and widely reported immunoregulatory effects of intermittent fasting [22,23,24,25,26], one recent study showed that MRL/lpr lupus-prone mice with intermittent fasting exhibited enhanced B cell activity and autoantibody production, and an exacerbated severity of nephritis [31]. The different impacts of intermittent fasting on B cell activity and function are probably attributed to distinct disease contexts, which awaits future investigations.

Patients with SS exhibit reduced PPARγ expression and impaired anti-inflammatory activity of PPARγ signaling in the salivary epithelia [32]. Accordingly, administration of PPARγ agonists suppressed lymphocytes infiltration and down-regulated Th1 cytokines in the salivary glands of NOD mice [33]. Using the same mouse model, our study demonstrated that ADF caused a remarkable increase in the mRNA levels of Cpt1a and Pdk4, two key target genes of the PPAR pathway, in the SMGs, which is consistent with other groups’ reports showing activation of PPAR signaling as one of the major biological processes elicited by fasting in multiple tissue types [34,35,36]. Taken together, PPARγ activation may mediate the attenuation of sialadenitis in NOD mice caused by the ADF regimen through dampening Th1 responses. In addition to Th1-mediated inflammation, PPARγ activation has been reported to mitigate Th17 responses in the central nervous system of mice with experimental autoimmune encephalomyelitis [37]. Whether PPARγ pathway is also involved in Th17-mediated autoimmunity in SS and whether PPARγ activation contributes to the attenuation of Th17 response by ADF in this disease-setting require future delineations.

We also found that ADF increased the mRNA levels of water channel protein AQP5 and tight junction protein claudin-1, two factors critical for normal salivary secretion [38,39,40,41,42]. Our previous study demonstrated that treatment of antibody clone targeting CXCR3 (CXCR3-173), which can suppress T cell migration by interrupting the interaction between CXCR3 and its ligands [43,44,45], led to an improvement in salivary secretion in NOD mice that was accompanied by a significant reduction in IFN-γ-producing CD8 T cells and an increase in the expression levels of AQP5 and claudin-1 in the SMGs [17]. Therefore, the improvement in the salivary gland secretory function by ADF could be, at least in part, attributed to an impaired CXCR3-dependent effector T cell migration to the SMGs and a consequent increase in AQP5 and claudin-1 expression levels. Meanwhile, the change of CXCR3 and key inflammatory cytokines in effector T cells, T cell-associated chemoattractants, AQP5 and claudin-1 in epithelial cells, at the protein level, in the SMGs induced by ADF requires further validation.

Female NOD mice have been reported to become diabetic around 12–16 weeks of age [46]. In the present study, none of the NOD mice exhibited abnormal urine glucose levels at 13 weeks of the age, the end-point analyses. Thus, the protective effect of ADF on sialadenitis and xerostomia in NOD mice during the early stage of SS establishment is unlikely an indirect consequence of its influence on diabetes. The influence of ADF on salivary disorders at later stages of SS awaits further investigation using other mouse models of this disease that do not develop diabetes.

## 4. Materials and Methods

### 4.1. Mice and Alternate-Day Fasting Intervention

Female non-obese diabetic (NOD) mice (the NOD/ShiLtJ strain) were purchased from the Jackson Laboratory (Bar Harbor, ME, USA) and maintained under specific pathogen-free condition at the Forsyth Institute. All experimental procedures were approved by the Institutional Animal Care and Use Committee of the Forsyth Institute and performed in compliance with the National Institutes of Health guidelines for the care and use of laboratory animals.

Mice in the ADF group were fed every other day, and age- and sex-matched mice fed standard chow ad libitum served as controls. All mice had unrestricted access to water throughout the entire experiments. A total of 12 mice per group were used in 4 independent experiments. The number of mice for each specific analysis was indicated in the responding figure legends.

### 4.2. Measurement of Stimulated Salivary Flow

Mice were intraperitoneally administered with 100 μL PBS-based secretagogue solution containing isoproterenol (1 mg/mL) and pilocarpine (2 mg/mL). One minute after the injection, saliva was collected continuously for 5 min from the oral cavity of mice with a micropipette. The measurement was consistently performed in the mid-afternoons to exclude the effect of circadian rhythm on the results.

### 4.3. Histologic Analysis

SMG tissues were harvested from euthanized mice and were fixed in 4% paraformaldehyde and embedded in paraffin. Five-micron sections were cut and stained with hematoxylin and eosin (H&E) to determine leukocyte infiltration of SMGs. The average numbers of leukocytic foci (comprising at least 50 mononuclear cells per 4 square millimeters) in each of three non-consecutive sections, separated by 100 microns were statistically analyzed, and the infiltration areas were measured with the Image-Pro Plus 6.0 software.

### 4.4. Flow Cytometry

Single cells isolated from SMGs or submandibular draining lymph node (smLN) cells were incubated with anti-CD16/32 antibody to block non-specific Fc receptor binding, and stained with a panel of fluorescence-conjugated antibodies against CD4, CD8, CD19, CD44, CD45 and CD62 at 4 °C for 30 min. For intracellular and nuclear staining, cells were then incubated with fluorescence-labeled anti-IFN-γ and anti-IL-17A antibodies following fixation and permeabilization. After being rinsed with cold PBS, the stained cells were analyzed with the FACS Arial II flow cytometer (BD, Franklin Lakes, NJ, USA) and FlowJo V10 software. All the antibodies utilized for flow cytometric staining were purchased from BioLegend (San Diego, CA, USA).

### 4.5. Real-Time PCR

Total RNA from SMGs and smLNs tissues were exacted using NucleoSpin RNA kit (Macherey-Nagel, Allentown, PA, USA) and then reverse transcribed into complementary DNA with MLV reverse transcriptase (Promega, Madison, WI, USA). The SYBR Green-based real-time PCR amplification was performed for 40 cycles with annealing and extension temperatures at 60 °C on a QuantStudio™ 3 Real-Time PCR System (Thermo Fisher Scientific, Waltham, MA USA). Primer sequences for mouse gene analyses are: GAPDH forward, 5′-AGGTCGGTGTGAACGGATTTG-3′, reverse, 5′-GGGGTCGTTGATGGCAACA-3′ IFN-γ forward, 5′-GGATGCATTCATGAGTATTGC-3′, reverse, 5′-CTTTTCCGCTTCCTGAGG-3′; TNF-α forward, 5′-CCTTTCACTCACTGGCCC AA-3′, reverse, 5′-AGTGCCTCTTCTGCCAGTTC-3′; IL-17 forward, 5′-GCGCAA AAGTGAGCTCCAGA-3′, reverse, 5′-ACAGAGGGATATCTATCAGGG-3′;

CXCR3 forward, 5′-GAGAGCGACTTCTCTGACTC-3′, reverse, 5′-ACTCAG TAGCACAGCAGCCA-3′; CXCL9 forward, 5′-CCCTCAAAGACCTCAAACAGT-3′, reverse, 5′-AGTCCGGATCTAGGCAGGTT-3′; CXCL10 forward, 5′- CCAGTG AGAATGAGGGCCAT-3′, reverse, 5′-CCGGATTCAGACATCTCTGC-3′; CXCL11 forward, 5′-GCAGAGATCGAGAAAGCTTCT-3′, reverse, 5′-GTCCAGGCACCT TTGTCGTT-3′. Other primer sequences are available upon request. The transcript levels were normalized to that of GAPDH.

### 4.6. Detection of Serum Antinuclear Antibodies (ANA)

Serum samples from mice were diluted 1:40 with cold PBS and subjected to ANA measurements using commercially available slides precoated with Hep-2 human epithelial cells (INOVA Diagnostics, San Diego, CA, USA), following the manufacturer’s instructions. The stained slides were imaged using an inverted wide-field fluorescence microscope (Zeiss, Dublin, CA, USA), and the fluorescence intensity of the staining was quantified with the ImageJ 1.50i software. The concentration of serum ANAs was also examined by ELISA with the Mouse ANA ELISA Kit (BIOMATIK, Wilmington, DE, USA), according to the manufacturer’s instruction.

### 4.7. Detection of Anti-M3 Muscarinic Acetylcholine Receptor (M3R)

The M3R peptide (AILFWQYFVGKRTVP) was synthesized by Biomatic Corporation and kindly provided by Dr. Toshihisa Kawai (Nova Southeastern University). The M3R peptide solution (1 µg/mL) was used to coat Nunc™ MaxiSorp™ flat-bottom 96-well plated (BioLegend) and incubated at 4 °C overnight. Non-specific binding sites on the plates were blocked with ELISA Assay Diluent buffer (BioLegend). Sera with serial dilution (1:80, 1:160, 1:320, 1:640, 1:1280, 1:2560, 1:5120) were added to the plate and incubated at 4 °C overnight. The plates were incubated with biotinylated goat anti-mouse IgG antibody (Vector Laboratories, Newark, CA, USA), Avidin-HRP solution and TMB substrate followed by reading as previously reported [28]. The absorbance was measured at 450 nm and 570 nm with a microplate reader (BioTek, Santa Clara, CA, USA), and the absorbance at 450 nm subtracted by that at 570 nm was considered as an indicator for anti-M3R antibody quantity.

### 4.8. Statistical Analysis

Statistical significance was determined by a two-tailed Student’s *t* test or ANOVA as appropriate. *p* values smaller than 0.05 were considered as statistically significant.

## 5. Conclusions

The present study demonstrates the beneficial impact of ADF on newly established SS-like sialadenitis and xerostomia in NOD mice, providing critical insight into a diet-based therapeutic strategy for an autoimmune exocrinopathy.

## Data Availability

Data sharing not applicable to this article as no datasets were generated or analyzed during the current study.
